# Pregnancy outcomes and risk factors for preeclampsia in dichorionic twin pregnancies after in vitro fertilization: a five-year retrospective study

**DOI:** 10.1186/s12884-022-05184-y

**Published:** 2022-11-10

**Authors:** Fen Dai, Shuangjia Pan, Yehui Lan, Hongying Tan, Jinman Li, Ying Hua

**Affiliations:** 1 Department of Obstetrics and Gynecology, the Second Affiliated Hospital of Wenzhou Medical University, Wenzhou, 325027 China; 2 Department of Obstetrics and Gynecology, Wenzhou Medical University, The Third People’s Hospital of Cangnan County, Wenzhou, China

**Keywords:** In vitro fertilization, Preeclampsia, Early-onset preeclampsia, Dichorionic twins, Risk factors

## Abstract

**Background:**

Both in vitro fertilization (IVF) and preeclampsia (PE) were associated with placental dysfunction. Although IVF can increase the incidence of PE, the pregnancy outcomes and risk factors for preeclampsia in dichorionic twin pregnancies conceived via IVF remain unclear. This study aimed to investigate the pregnancy outcomes and the risk factors for preeclampsia in dichorionic twin pregnancies conceived through IVF compared to those conceived after natural conception (NC).

**Methods:**

This retrospective observational study enrolled 181 dichorionic twin pregnancy women with preeclampsia from 2016 to 2020. According to the mode of conception, they were allocated into IVF (*n* = 117) and NC groups (*n* = 64). The clinical characteristics of preeclampsia and pregnancy outcomes between the two groups were compared by using Student’s t test, chi-square test, and Fisher’s exact test, and logistic regression models were used to obtain adjusted odds ratios (aOR) with 95% confidence intervals (CI) for risk factors of early-onset preeclampsia.

**Results:**

The incidence of early-onset PE and growth discordance in dichorionic twin pregnancies with PE is significantly higher in IVF-PE group than in NC group (78.60% vs 43.80%, *P* < 0.001, 11.10% vs 25.00%, *P* = 0.015). We found that IVF (aOR = 4.635, 95% CI: 2.130–10.084, *P* < 0.001) and growth discordance (aOR = 3.288; 95% CI: 1.090–9.749, *P* < 0.05) increased the incidence of early-onset PE.

**Conclusions:**

In preeclamptic dichorionic twin pregnancies, IVF and growth discordance were associated with the increased incidence of early-onset PE. The underlying mechanism for the relationship between IVF and early-onset PE or growth discordance may be placental dysfunction.

## Background

Preeclampsia (PE), characterized by new-onset maternal hypertension with end organ injury and proteinuria after 20 weeks of gestation, and was divided into early-onset (< 34 weeks) and late-onset preeclampsia (≥34 weeks) according to the time of onset PE [1, 2], especially early-onset preeclampsia has substantial contributions to severe obstetric adverse complications such as postpartum hemorrhage, fetal growth restriction, preterm birth and long-term cardiovascular disease (CVD) in the mothers [3, 4]. It affects approximately 3 to 5% of all pregnancies worldwide [1]. In China, PE accounted for 4.02–5.22% of all pregnancies, of which mild preeclampsia accounted for 15.13–17.00% and severe preeclampsia accounted for 36.30–39.96% [5, 6]. PE is the leading cause of maternal death in industrialized countries, and low-income countries may suffer from a higher burden of maternal death due to lack of access to adequate obstetric care [7, 8].

However, the etiology of this heterogeneous syndrome is not completely understood, although many risk factors for PE, such as advanced maternal age, multiple pregnancy, assisted reproductive technology, pregestational hypertension and diabetes have been identified [9]. There is accumulating evidence that variable degrees of placental malperfusion was the pathogenesis of pre-eclampsia [1, 4]. and in vitro fertilization (IVF) was also strongly associated with ischemic placental disease [10]. In view of the similar placental pathologic mechanisms, IVF has been proved as an independent risk factor for PE [9].

In recent years, with the extensive introduction and application of assisted reproductive technology mainly IVF, the incidence of twin pregnancy has risen sharply. Women who conceived twins through IVF had an increased risk of hypertensive disorders of pregnancy (HDP) and PE [11, 12]. A recent study analyzed the clinical characteristics and pregnancy outcomes of preeclampsia women with twins via IVF, and found no significant differences in rates of severe PE, early-onset PE, as well as mean systolic, or diastolic blood pressure compared to preeclamptic women conceiving twins naturally [13]. But it remained a challenge to obtain conclusive evidence because the sample size was small and their study did not calculate the risk ratio. Moreover, perinatal complications, for example, intrahepatic cholestasis of pregnancy (ICP) and growth discordance, the latter being unique in twin pregnancies, were associated with an increased risk for severe and early-onset PE [14, 15]. Hence, it is more scientific to evaluate the effect of IVF on PE or the early-onset PE when taking these variables into account.

The purpose of this study was to investigate differences in clinical manifestations and pregnancy outcomes in preeclampsia women with twins after IVF compared with natural conception, and further explore the association of IVF with early-onset preeclampsia and the potential risk factors for early-onset preeclampsia after adjustment for confounders.

## Methods

### Study design and subjects

This retrospective observational study enrolled all the women with twin pregnancies who were monitored prenatally and delivered in the Second Affiliated Hospital of Wenzhou Medical University, a maternal-fetal medicine center with 8000–10,000 births per year, between January 2016 and December 2020. Inclusion criteria were: dichorionic twin pregnancies with preeclampsia and nonsmoking Han Chinese. The exclusion criteria were: (1) preeclampsia in a previous pregnancy, patients complicated with diseases such as chronic hypertension or development of superimposed PE (PE in addition to baseline chronic hypertension), gestational hypertension, pregestational diabetes, kidney disease, autoimmune disease; (2) one fetus with intrauterine death or multifetal pregnancy reduction (MFPR); (3) fetal congenital anomalies and pregnancies resulting in miscarriage; (4) mono-chorionic twins; (5) pregnancies conceived by ovulation induction and intrauterine insemination. Participants in this study were divided into IVF group and the NC group according to the mode of conception.

### Ethical statement

The study protocol was reviewed and approved by the medical ethics committee of The Second Affiliated Hospital of Wenzhou Medical University. Women were informed that their records could be used to evaluate medical practices and were allowed to opt out of the study.

### Data collection

Data were obtained from medical records and was independently reviewed by an experienced obstetrician, including the mode of conception, demographic features, clinical characteristics of PE, laboratory values, obstetric complications and neonatal outcomes. The clinical indicators of PE included maximum systolic/ diastolic BP, the percentage of systolic BP (blood pressure) ≥ 180 mmHg or diastolic BP ≥ 120 mmHg, 24-hour urine protein, eclampsia, HELLP syndrome, early-onset PE and severe PE, as well as some specific symptoms of severe PE such as pleural effusion and (or) ascites, liver or (and) renal insufficiency, thrombocytopenia and cardiac failure. Maternal outcomes included mode of delivery, the gestational age at delivery, gestational diabetes mellitus (GDM), gestational hypothyroidism, ICP, postpartum hemorrhage (PPH), placental abruption and oligohydramnios. Neonatal outcomes included growth discordance in twins, intrauterine growth retardation, neonatal birth weight, very low birth weight and neonatal asphyxia.

### Relative definitions

The definition of PE and eclampsia were based on the current criteria from ACOG (American College of Obstetrics and Gynecology) [9]. PE was subdivided into mild and severe PE according to the signs and symptoms of PE, and early-onset PE and late-onset PE based on onset time of PE [9]. Chorionicity and amnionicity were diagnosed by ultrasound between 7 and 14 weeks’ gestation and confirmed by postnatal placental pathology. Growth discordance between twins was defined as a twin birth weight difference ≥ 20% and calculated by the following equation (large fetal birth weight-small fetal birth weight) / large fetal birth weight × 100%) [16].

GDM was diagnosed by a 75 g oral glucose tolerance test according to International Association of Diabetes in Pregnancy Study Groups (IADPSG) criteria [17]. The diagnose of intrahepatic cholestasis of pregnancy (ICP) was established in presence pruritus and elevated bile acids [18]. Gestational hypothyroidism was diagnosed when TSH levels > 2.5mIU/L in the first trimester or 3.0mIU/L in the second and third trimester [19]. Additional information extracted in detail from the medical records is as follows: preterm birth (birth at < 37.0 week’s gestation) and very preterm birth (birth at < 34 week’s gestation), postpartum hemorrhage (PPH, blood loss more than 1000 ml in 24 hours by Caesarean section or > 500 ml via vaginal delivery), neonatal asphyxia (5-minute Apgar score < 7),, intrauterine growth restriction (IUGR) was diagnosed when only one fetus’s birthweight was below the 10th percentile according to the Chinese twin reference curve [20]. very low birth weight (birth weight < 1500 g), placental abruption (premature separation of a normally implanted placenta before birth).

### Statistical analysis

Data analysis was performed using SPSS version 22.0 (SPSS, Statistical Package for the Social Sciences, IBM, NY, USA). The continuous data with normal distributions were presented as mean and standard deviation (SD), while categorical variables were expressed as numbers and percentages (%). Parametric t-tests, Chi-square tests and Fisher’s exact test were used to compare the demographic characteristics in dichorionic twins by IVF and those by NC according to the feature of variables. Logistic regressions were used to model the associations between early-onset PE and late-onset PE to identify potential risk factors. The odds ratio (OR) and 95% confidence interval (CI) after adjustment for potential confounders such as maternal age, body mass index (BMI) at delivery, gravidity, primipara, IVF, growth discordance and ICP were calculated. A 2-tailed *P* < 0.05 was considered statistically significant.

## Results

**Fig. 1:**
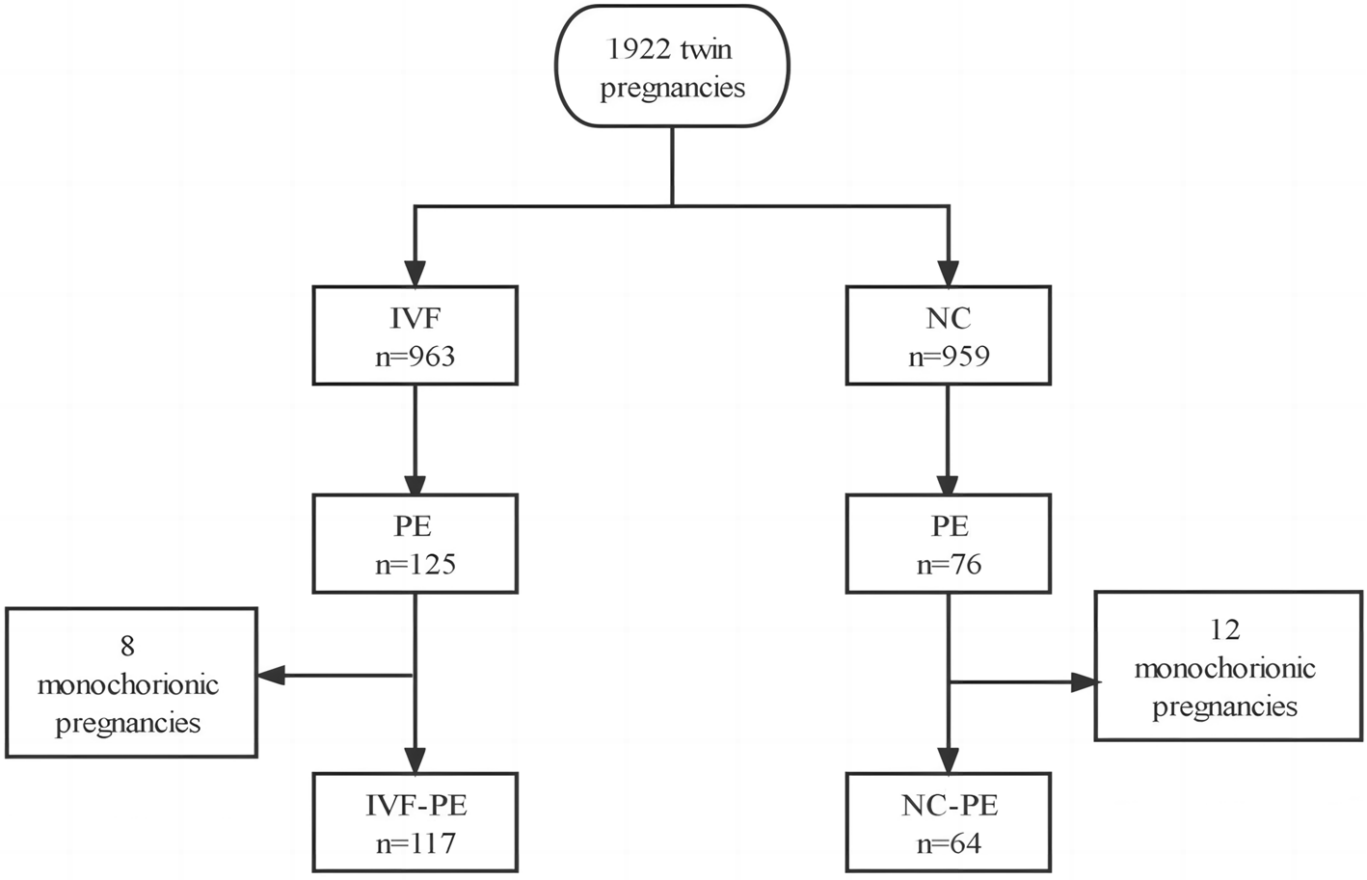
Flowchart of participants in the study

During the study period from 2016 to 2020, 1922 women with twin pregnancies gave birth in our hospital. Of these, 963 (50.10%) conceived via IVF and 959 (49.90%) conceived via natural conception. The incidence of PE was higher in women who conceived twins after IVF compared with those conceived naturally (OR 1.733; 95%CI, 1.283–2.340). After exclusion of 20 women with monochorionic pregnancies, 181 twin pregnancies with PE remained and constituted the study group: 117 pregnancies resulted from IVF and 64 pregnancies from NC (Fig. 1).

### Patient population

The maternal baseline characteristics were analyzed in Table 1.Women in the IVF-PE group were significantly older than those who conceived naturally (30.96 ± 3.88 vs 28.63 ± 3.45; *P* < 0.001). And the BMI at delivery was higher in IVF-PE group than NC-PE group (29.31 ± 3.69 vs 28.14 ± 3.15; *P* < 0.05). Also, we found higher rates of primipara, pregestational obesity and low-dose aspirin (LDA) use in IVF-PE group versus NC-PE group (*P* < 0.05). The groups did not differ significantly in gravidity, pregnancy weight gain, the percentage of advanced age and family history of preeclampsia.

**Table 1:** Maternal characteristics of IVF-PE and NC-PE group

	IVF-PE group (*n* = 117)	NC-PE group (*n* = 64)	*P* value
Maternal age (years)	30.96 ± 3.88	28.63 ± 3.45	**0.000**
Advanced age (≥35 years) n (%)	21 (17.9)	15 (23.4)	0.376
BMI at delivery (kg/m2)	29.31 ± 3.69	28.14 ± 3.15	**0.033**
Gravidity n (%)			0.050
1	52 (44.5)	33 (51.5)	
2	37 (31.6)	14 (21.9)	
3	15 (12.8)	3 (4.7)	
≥ 4	13 (11.1)	14 (21.9)	
Parity n (%)			**0.021**
0	101 (86.3)	44 (68.7)	
1	13 (11.1)	16 (25.0)	
≥ 2	3 (2.6)	4 (3.3)	
Pregestational obesity n (%)	15 (12.8)	2 (3.1)	**0.033**
Pregnancy weight gain (kg)	16.22 ± 2.14	16.63 ± 1.78	0.178
Family history of preeclampsia n (%)	7 (6.0)	5 (7.8)	0.872
LDA usage n (%)	24 (20.5)	5 (7.8)	**0.026**

All data are expressed as the mean ± standard deviation or n (%)

*Abbreviations*: *IVF* In vitro fertilization, *NC* Natural conception, *PE* Preeclampsia, *LDA* Low-dose aspirin

Clinical characteristics, perinatal complications and outcomes were summarized in Table 2. Compared with preeclamptic dichorionic twin pregnancies after natural conception, the incidence of early-onset PE and growth discordance is significantly higher in women after IVF. Of course, the gestational weeks at diagnose of PE is earlier in IVF-PE group. Other clinical characteristics, including gestational weeks at delivery, hospitalization time, systolic BP, diastolic BP, urine protein, the rate of severe PE, HELLP syndrome, placental abruption and end-organ dysfunction were similar between two groups. No significant difference was found between IVF-PE and NC-PE groups regarding the rate of perinatal complications and outcomes (*P* > 0.05).

**Table 2:** Clinical characteristics and pregnancy outcome of IVF-PE and NC-PE group

	IVF-PE group (*n* = 117)	NC-PE group (*n* = 64)	*P* value
Gestational age at delivery (weeks)	35.27 ± 2.05	35.21 ± 2.24	0.853
Gestational age at diagnosis (weeks)	32.68 ± 2.64	33.76 ± 2.58	**0.009**
Hospitalization time (days)	9.44 ± 5.57	8.94 ± 3.71	0.474
Maximum systolic BP, mmHg	163.17 ± 12.06	163.63 ± 10.24	0.799
Maximum diastolic BP, mmHg	102.99 ± 7.74	101.64 ± 6.69	0.241
Systolic BP ≥ 180 mmHg n (%)	12 (10.2)	6 (9.4)	0.850
Diastolic BP ≥ 120 mmHg n (%)	3 (2.6)	0 (0)	0.495
24-hour urine protein (g)	2.53 ± 3.40	2.09 ± 2.44	0.354
Severe PE n (%)	97 (82.9)	48 (75.0)	0.203
Early-onset PE n (%)	92 (78.6)	28 (43.8)	**< 0.001**
Pleural effusion and (or) ascites n (%)	22 (18.8)	10 (15.6)	0.592
Liver or (and) renal insufficiency n (%)	14 (12.0)	8 (12.5)	0.916
Thrombocytopenia n (%)	10 (8.5)	4 (6.3)	0.793
Placental abruption n (%)	10 (8.5)	2 (3.1)	0.276
HELLP syndrome n (%)	8 (6.8)	2 (3.1)	0.481
Eclampsia or Cardiac failure n (%)	5 (4.3)	3 (4.7)	1.000
Maternal complications			
Gestational diabetes mellitus n (%)	28 (23.9)	14 (21.9)	0.754
Ggestationa hypothyroidism n (%)	13 (11.1)	6 (9.4)	0.716
Intrahepatic cholestasis during pregnancy n (%)	13 (11.1)	7 (10.9)	0.972
Postpartum hemorrhage n (%)	20 (17.1)	6 (9.4)	0.157
Oligohydramnios n (%)	5 (4.3)	4 (6.3)	0.820
Growth discordance in twins n (%)	13 (11.1)	16 (25.0)	**0.015**
Intrauterine growth retardation n (%)	35 (29.9)	24 (37.5)	0.298
Perinatal outcome			
Birth weight (g)	2266.26 ± 498.66	2212.97 ± 499.93	0.493
Caesarean section n (%)	113 (96.6)	60 (93.8)	0.612
Preterm delivery n (%)	64 (54.7)	35 (54.7)	0.999
Very preterm delivery(< 34 weeks) n (%)	25 (21.4)	14 (21.9)	0.937
Very low birth weight n (%)	11 (9.4)	8 (12.5)	0.516
Neonatal asphyxia n (%)	64 (54.7)	38 (59.4)	0.544

All data are presented as the mean ± standard deviation or n (%)

*Abbreviations*: *IVF* In vitro fertilization, *NC* Natural conception, *PE* Preeclampsia, *BP* Blood pressure, *HELLP* Syndrome hemolysis, elevated liver enzymes, and low platelet count

**Fig. 2:**
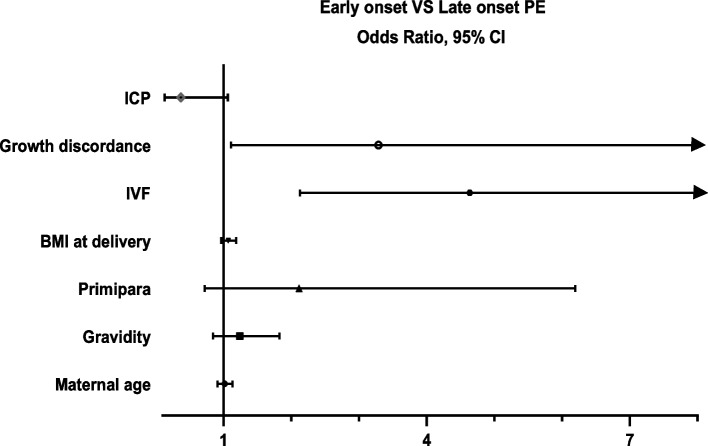
Forest plot of risk factors for early-onset preeclampsia

Compared to naturally conceived pregnancies, the odds ratios of early-onset PE were significantly increased in the IVF group (OR 4.731; 95% CI:2.439–9.180, *P* < 0.001), with an adjusted odds ratio (aOR) of 4.635 (95% CI: 2.130–10.084, *P* < 0.001) after adjustments for confounding factors, including ICP and growth discordance. Moreover, the growth discordance appeared to be associated with an increased risk for early-onset PE (aOR = 3.288; 95% CI: 1.109–9.749, *P* < 0.05) after accounting for confounding factors (Table 3 and Fig. 2).

**Table 3:** Logistic regression analysis of risk factors for early-onset PE

Risk factors	Univariate		Multivariate		*P* value
	OR	95% CI	aOR^a^	95% CI	
Maternal age	1.083	0.996–1.177	1.016	0.910–1.134	0.784
Gravidity	1.178	0.893–1.553	1.241	0.844–1.825	0.271
Primipara	1.537	0.727–3.252	2.117	0.723–6.196	0.171
BMI at delivery	1.102	1.001–1.210	1.070	0.966–1.185	0.194
IVF	4.731	2.439–9.180	4.635	2.130–10.084	< 0.001
ICP	0.369	0.144–0.945	0.371	0.130–1.061	0.064
Growth discordance	1.732	0.695–4.316	3.288	1.109–9.749	0.032

*Abbreviations*: *PE* Preeclampsia, *OR* Odds ratio, *aOR* Adjusted odds ratio, *CI* Confidence interval, *BMI* Body mass index, *LDA* Low-dose aspirin, *IVF* In vitro fertilization, *ICP* Intrahepatic cholestasis of pregnancy

^a^Adjustments for maternal age, BMI at delivery, gravidity, primipara, ICP, IVF and growth discordance

## Discussion

In this study, we found that PE was more common in dichorionic twin pregnancies via IVF than via natural conception. In addition, we observed a higher rate of early-onset PE and growth discordance in IVF-PE group than NC-PE group. Last but most importantly, in preeclamptic dichorionic twin pregnancies, IVF and growth discordance seem to increase the risk for early-onset PE after adjusting for various confounders.

Our finding that the rate of PE was higher in IVF twin pregnancies supported the previous studies [13, 21]. Although interest in this area is growing rapidly, it’s not clear whether IVF itself has an impact on the occurrence of PE. Several pathophysiological hypotheses have been proposed to explain the relationship between IVF and PE. One of the strongest possibilities is that the IVF process itself is associated with epigenetic changes and gene expression changes in fetal and maternal tissues, and that modifications of the maternal endometrium can cause trophoblast infiltration and placental dysfunction, triggering placental diseases in early pregnancy [22, 23]. These findings also were confirmed in some studies that IVF increase ischemic placental diseases [10].

One of the important evidences is that angiogenic factors produced by the placental tissue are involved in endothelial injury and vasoconstriction, leading to placental dysfunction, such as soluble fms-like tyrosine kinase-1 (sFlt-1) and soluble endoglin serum levels are significantly elevated in patients with early-onset preeclampsia (< 34 weeks) [24]. Furthermore, in the first trimester, serum concentrations of sFlt-1 were higher in twin pregnancies conceived by ART than in spontaneous twin pregnancies [25].

Another evidence was that patients with early-onset PE had higher of alpha fetoprotein (AFP) value at their second trimester biochemical screening [26], and the levels of AFP were significantly higher in IVF pregnancies versus natural conception pregnancies [23, 27]. Early-onset PE arises primarily due to defective placentation during the first few weeks of pregnancy [28]. Therefore, these evidences suggest that IVF itself may play a role in the increased incidence of early-onset PE.

The clinical manifestations of PE is highly variable among individuals. Our study found that twin pregnancies via IVFwere more likely to develop an early-onset PE and this risk remained after adjustment for confounders Interestingly, in spite of the increasing occurrence of early onset PE, the gestational age at delivery and the rate of preterm were not significantly different between IVF-PE group and NC-PE group. One potential explanation was that the enhanced awareness of antenatal care among the IVF pregnancies, as well as great attention from obstetricians, may play a protective role in these pregnancy outcomes.

Growth discordance, defined as a difference of 20% in the birth weights between twins, is a unique complication of multiple pregnancies. In dichorionic twins, each fetus has its own placenta and vascular anastomoses, and the growth curve is theoretically similar to that of a singleton fetus before 32 weeks [29]. But a large, prospective, multicenter study reported that the rate of the placental abnormalities in both birthweight discordance and small for gestational age were significant higher in dichorionic twins versus monochorionic twins [30]. IVF procedure is associated with placental abnormalities [31]. This may account for our findings that the proportion of growth discordance in dichorionic twin pregnancies was higher in the IVF-PE group, which is corroborate with previous data [32]. Another plausible explanation for this result is that IVF pregnancies appear to have a higher proportion of genetic dissimilarity and different genetic growth potentials, resulting in the discordant growth [33].

On the other hand, growth discordance is associated with a high risk for preeclampsia in dichorionic twin pregnancy [15, 34]. Sharing the same pathophysiologic mechanism may explain the interaction between preeclampsia and growth discordance.. Placental ischemia/hypoxia, which coexist either in preeclampsia or growth discordance women, appears to be responsible for this association [24]. This association is further supported by our findings that growth discordance is strongly associated with early-onset PE after adjusting for confounders.

Furthermore, common umbilical cord abnormalities, such as velamentous cord, may mediate the association between growth discordance and PE in twin pregnancies. Velamentous cord insertions, a major indicator of growth discordance, has been reported as a risk factor for PE [35]. In addition, advanced maternal age was associated with an increased incidence of severe growth discordance and PE [9, 36], which may be another risk factor. Based on these findings, we speculate that the relationship between growth inconsistency and PE may not be caused by a single cause, but by the coexistence of multiple factors.

PE and IVF share multiple risk factors such as advanced maternal age, nulliparity, and obesity, which were indeed significantly different between the IVF group and the spontaneous group in previous studies [12, 22]. Older mothers or obese women may be at risk for endothelial dysfunction, making them more susceptible to pre-eclampsia, and these women often conceive the baby via IVF.. Likewise, our data showed that in twin pregnancies with PE, IVF was associated with older maternal age, higher BMI at delivery, higher rates of primipara and pregestational obesity, and higher use of low-dose aspirin (LDA) compared with natural conception. But advanced maternal age, obesity and primipara were not risk factors for early-onset preeclampsia. The similar results were found in the previous studies [37, 38].

In addition, low-dose aspirin (LDA) can effectively reduce the incidence of severe PE [39]. The underlying mechanism is that LDA can improve spiral artery blood flow, promote trophoblast migration and subsequent spiral artery remodeling, thereby preventing placental defects. Nevertheless, once preeclampsia has developed, aspirin does not seem to change the course of the disease because the insufficient placenta has been formed [40]. National guidelines also recommend commencing aspirin as soon as possible after the end of early pregnancy to prevent preeclampsia [9]. However, our research indicated that despite higher aspirin use in the IVF-PE group, there was no difference in the incidence of severe PE compared to the NC-PE group. Aspirin use appeared to have no benefit in reducing the incidence of severe PE.It is difficult to explain. We proposed a hypotheses that preconception or early administration of low-dose aspirin may not ameliorate the placental dysfunction which is closely associated with the early-onset PE. In addition, it has also been proposed that the administration of low-dose aspirin at < 11 weeks’ gestation in high-risk women, including those pregnancies conceived via IVF, does not reduce the risk of preeclampsia, gestational hypertension and any hypertensive disorder of pregnancy [41]. It has been believed that IVF, at least in part, was responsible for increasing the incidence of severe preeclampsia [42, 43]. Further research is needed to clarify this mechanism.

The strengths of the present study is that we focused on the dichorionic twin pregnancies in order to reduce the confounding, as chorionicity was an independent risk factor for PE and early-onset PE [44], and we excluded the confounders such as pre-gestational diseases, immune system disorders, and intrauterine treatment. Futhermore, we assessed risk factors for early-onset preeclampsia, especially after adjusting for confounding variables such as baseline characteristics and pregnancy complications.

The limitations of our study is a single-center, retrospective and small sample size analysis, which limites the generalizability/power of findings. Large prospective trials and the long term follow up are needed to investigate the effect of IVF on the pregnancy outcomes and the risk factors of preeclampsia in dichorionic twin pregnancies.

## Conclusions

Overall, in dichorionic twin pregnancies with pre-eclampsia, IVF increase the incidence of early-onset PE and act as an isolated risk factor. Furthermore, growth discordance appears to be associated with the higher incidence of early-onset PE. Placental abnormalities maybe responsible for this association. Of course, due to the co-existence of individual variability and confounding bias associated with pre-eclampsia, it warrants further investigation.

## Data Availability

The datasets used and/or analyzed during the study are available from the corresponding author upon reasonable request.

## Abbreviations

IVF: In vitro fertilization
PE: Preeclampsia
NC: Natural conception
aOR: Adjusted odds ratios
CI: Confidence intervals
CVD: Cardiovascular disease
HDP: Hypertensive disorders of pregnancy
ICP: Intrahepatic cholestasis of pregnancy
MFPR: Multifetal pregnancy reduction
BP: Blood pressure
HELLP: Hemolysis, elevated liver enzymes, and low platelet
GDM: Gestational diabetes mellitus
PPH: Postpartum hemorrhage
ACOG: American College of Obstetrics and Gynecology
SPSS: Statistical Package for the Social Sciences
SD: Standard deviation
OR: Odds ratios
BMI: Body mass index
LDA: Low-dose aspirin
sFlt-1: Soluble fms-like tyrosine kinase-1
